# Probiotic *Saccharomyces cerevisiae* var. *boulardii* supernatant inhibits survivin gene expression and induces apoptosis in human gastric cancer cells

**DOI:** 10.1002/fsn3.2032

**Published:** 2020-11-29

**Authors:** Babak Pakbin, Shaghayegh Pishkhan Dibazar, Samaneh Allahyari, Maryam Javadi, Alireza Farasat, Sina Darzi

**Affiliations:** ^1^ Department of Food Hygiene and Quality of Control Faculty of Veterinary Medicine University of Tehran Tehran Iran; ^2^ Department of Immunology Faculty of Medical Science Tarbiat Modares University Tehran Iran; ^3^ Department of Food Hygiene and Safety School of Health Qazvin University of Medical sciences Qazvin Iran; ^4^ Children Growth and Development Research Center Research Institute for prevention of Non‐ Communicable Disease Qazvin University of Medical Sciences Qazvin Iran; ^5^ Cellular and Molecular Research Center Research Institute for prevention of Non‐Communicable Disease Qazvin University of Medical Sciences Qazvin Iran; ^6^ Department of Medical Biotechnology Qazvin University of Medical Sciences Qazvin Iran; ^7^ Health Products Safety Research Center Qazvin University of Medical Science Qazvin Iran

**Keywords:** apoptosis, gastric cancer cells, *Saccharomyces boulardii* supernatant, survivin gene expression

## Abstract

Natural anticancer drug and compounds with other great benefits are of interest recently due to lower side effects than chemotherapy for cancer treatment and prevention. Different natural and synthetic drugs have been suggested to be used for treatment of gastric cancers, the second deadly cancer worldwide. The aim of this study was to investigate anticancer activity of SBS including inducing apoptosis and inhibition of survivin gene expression in gastric cancer cells. We evaluated cell viability, inducing apoptosis and change in survivin gene expression of EPG85‐257P (EPG) and EPG85‐257RDB (resistant to Daunorubicin, RDB) cell lines under exposure of SBS after 24, 48, and 72 hr. We found that SBS decreased cell viability, induced apoptosis, and reduced survivin gene expression in treated EPG and RDB cells (with the significant IC_50_ values of 387 and 575 µg/ml after 72 and 48 hr for EPG and RDB cells respectively). However, we observed SBS was more efficient to induce apoptosis in EPG than RDB cells. We strongly suggest SBS be considered as a prospective anticancer agent or in formulation of complementary medication to treat and prevent gastric cancers.

## Graphical abstract

We found that SBS decreased cell viability, induced apoptosis, and reduced survival gene expression in treated EPG and RDB cells. However, we observed SBS was more efficient to treat EPG than RDB cells. We strongly suggest SBS be considered as a prospective anticancer agent or in formulation of food functional additives to treat and prevent gastric cancers.

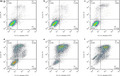

## 1 INTRODUCTION

Gastric cancer, the fourth most prevalent and the second deadly cancer worldwide, has been dramatically predominant in the developing countries. The incidence of this chronic disease has daily been increasing in the developed countries (Van Cutsem et al., 2016). The etiology of gastric cancer contributes to some major risk factors consisting of *Helicobacter pylori* infection, dietary factors, tobacco consumption, and obesity (Thrift & El‐Serag, 2020). There are two main strategies against gastric cancer, prevention and treatment. Obviously, early detection and prevention methods are more recommended as the superior strategies, but in several cases, treatment of patients is the only possible solution (Song et al., 2017). There are many types of treatments developed to improve patients with gastric cancer such as surgical treatment, radiotherapy, chemotherapy, chemoradiotherapy, and targeted therapy. Routine treatment recently been employed for gastric cancer patients is surgery while adjuvant chemotherapy; however, this method remains many lethal side effects on patients (Wagner et al., 2017). Despite significant advances in development of different treatment and drugs with lower side effects, researchers have still shined the light on drug formulated with natural compounds to provide more efficient treatment (Charalampakis et al., 2018).

Probiotics are viable microorganisms and beneficially affect the gastrointestinal system of human and animals providing some health promotion effects such as prevention of pathological conditions, balancing intestinal flora, stimulation of immune system, treatment of GI disorders, antioxidant, and anticancer activities (Cremon et al., 2018). Probiotics consist of some bacterial and fungal strains such as *Saccharomyces cerevisiae* var. *boulardii* (*S. boulardii*) as a yeast probiotic. *S. boulardii* is one of the varieties of *S. cerevisiae* strains providing probiotic activity (Czerucka et al., 2007). *S. cerevisiae* are practical and industrial yeast used as fermentative starter in bakery products. Some varieties of this yeast are opportunistic pathogens led to fungemia in human and animals; on the other hand, probiotic and functional activities of *S. cerevisiae* var. *boulardii* have been approved. Due to pathogenic probability of *S. boulardii*, many researchers have recently recommended SBS to exploit probiotic and health promotion benefits (Appel‐da‐Silva et al., 2017). Supernatant of yeast has been employed for enrichment of dairy cattle feeding. Major component of *S. boulardii* supernatant (SBS) is some polysaccharides such as different types of D‐glucans, chitin, and mannoproteins. Interaction with immune response, prevention of pathogen colonization, pathogen mitigation, antioxidant, anti‐inflammatory, antitumor, and antineoplastic properties of SBS mainly associate with these functional polysaccharides (Fortin et al., 2018b). Many studies investigated the health promotion effects of different components in yeast supernatant and SBS. They reported that compounds under 10 KD molecular weight such as β‐glucans contribute to anticancer and antioxidant activities (Fortin et al., 2018a). Regarding many studies proved anticancer effects of SBS on cancer cells, these properties of SBS have not been investigated on human gastric cancer cells. Thus, the main aims of the present study were to investigate apoptosis and survivin gene expression in human gastric cancer cells treated with SBS.

## 2 MATERIALS AND METHODS

### 2.1 *S. boulardii* supernatant preparation

For *Saccharomyces boulardii* supernatant (SBS) preparation, lyophilized probiotic *S. cerevisiae* var. *boulardii* CNCM I‐745 (Yomogi_®_, Mutaflor Co., Australia) purchased from a local drug store in Qazvin, Iran, was used. Lyophilized yeast was directly mixed with RPMI 1640 (Gibco, Thermo Fisher Scientific, USA) cell culture medium supplemented with 10% (v/v) FBS (fetal bovine serum, Gibco, Thermo Fisher Scientific, USA) and antibiotics (100 µl/ml streptomycin and 100 µl/ml penicillin; Gibco, Thermo Fisher Scientific, USA) and then incubated for 24 hr at 37°C. After incubation, the suspension was centrifuged for 15 min at 6300 *g*; then, the supernatant was collected. To the removal of yeast cells residual, the supernatant was passed through 0.22‐µm filters (Sigma‐Aldrich, MilliporeSigma Co., Germany); then, the filtrate was considered as the SBS treatment. SBS was freeze‐dried and diluted in 250, 500, 750, 1,000, 1,250, and 1,500 µg/ml concentrations with cell culture medium supplemented with FBS and antibiotics for treatment of cancer cells.

### 2.2 Cell cultures and treatments

Human adenocarcinoma gastric cell lines consisting of EPG85‐257P (EPG) and EPG85‐257RDB (RDB) were purchased from Pasteur Institute (Pasteur In., Iran) and used as the cell models. All cell lines were activated in RPMI 1640 cell culture medium supplemented with 10% (v/v) FBS and antibiotics (100 µl/ml streptomycin and 100 µl/ml penicillin) with incubation at 37°C and 5% CO_2_. Stock cells were passaged every week during the experiments. Subculture of the stock cells was prepared for conducting anticancer treatments by cell culturing into the 96‐well microplates at 80% confluence. After 48 hr and formation of cell monolayer in each well, all cells were treated with SBS. Treated cells with SBS dilutions and the control sample (including cells treated with DMSO) were harvested for viability assessment by MTT assay, total RNA extraction for gene expression measurement by real‐time PCR, and cell apoptosis analysis by flow cytometry after 24, 48, and 72 hr.

### 2.3 Cell viability assessment

Viability of the treated cancer cells was assessed using MTT assay. 3‐(4,5‐dimethylthiazol‐2‐yl)‐2,5 diphenyl tetrazolium bromide (MTT) was employed for evaluation of viability of treated cancer cells. For carrying out MTT assay, 96‐well microplate containing treated EPG or RDB cells was renewed with RPMI 1640 medium containing 0.5 mg/ml MTT and then incubated at 37°C and 5% CO_2_ for 4 hr. After discarding the medium, dimethyl sulfoxide (DMSO) was added into each well. MTT (yellow) is reduced to formazan (purple at 570 nm absorbance) through the enzymatic reaction in wells containing viable cells. Microplate reader model Elx808 (BioTek, USA) was used to measure the absorbance of the microplate and evaluate the viable cells in each well. Cell viability percentage was measured using the following formula:

$$\text{Cell}\;\;\text{viability}\;{\text{(\textbackslash{}\% ) = A}}_{e}‐A_{n}/A_{c}‐A_{n}\times 100$$

While A_n_ is the absorbance of the blank, A_e_ is the absorbance of the experiment and A_c_ is the absorbance of the control. Inhibitory concentrations of 50% (IC_50_) were calculated as the SBS dilution decreases the cell viability of the cells seeded in each well to 50% in comparison with the untreated sample (Śliwka et al., 2016). SBS with IC_50_ value concentration was used to treat the cancer cells following apoptosis and gene expression analysis.

### 2.4 Cell apoptosis analysis

Apoptosis of the treated cancer cells was analyzed using flow cytometry method. EbioScience cell apoptosis kit (Ebioscience, San Diego, USA) containing propidium iodide (PI) and Annexin V‐FITC staining and FACS‐Calibur flow cytometer machine (Dickinson Immunocytometry system, CA, USA) was used to evaluate the apoptosis of treated EPG and RDB cells. As described in the manufacturers` instructions of the cell apoptosis kit, first 10^6^ cells per well were seeded in a 6‐well microplate, then treated with IC_50_ value concentration of SBS, and incubated at 37°C and 5% CO_2_ for 24, 48, and 72 hr. Then, cells (treated and control or untreated) were harvested and washed with phosphate‐buffered saline (PBS). Harvested cells were incubated with PI and Annexin V‐FITC for 5 and 30 min, respectively, in a dark place at room temperature. After staining, expression of PI and annexin was measured using the flow cytometry instrument.

### 2.5 Gene expression measurements

For assessment of anticancer effect of SBS on treated EPG and RDB cells, expression of survivin mRNA was measured. Expression of survivin gene in treated (with IC_50_ value concentration of SBS) and control (untreated) EPG and RDB cells was evaluated using reverse transcriptase real‐time PCR coupled to 2^‐ΔΔCt^ method. First, total RNA of harvested cells was extracted employing RiboEx total RNA extraction kit (GeneAll Biotechnology Co., Korea) according to manufacturers’ instructions. ABI PCR thermal cycler machine model 9092 (Applied biosystems, USA) and GeneAll cDNA synthesis kit (GeneAll Biotechnology Co., Korea) were used to reverse transcribe the extracted RNA according to the kit manufacturers’ instructions. Real‐time PCR was performed using qRT‐PCR SYBR green ROX supermix (Ampliqon, Denmark) with the RotorGene real‐time PCR machine 6000 (Qiagen, USA). Each reaction tube contained 25 µl reaction mix consisting of 12.5 µl supermix kit, 0.5 µl of each primer (10 µM/µl), and 1 µl cDNA template and sterilized deionized water to the final reaction volume. GAPDH (glyceraldehyde 3‐phosphate dehydrogenase) primers were used as the internal control. Primer sequences synthesized by SinaColon (SinaColon Co., Iran) are described as follows: Survivin forward 5′‐ATG GCA CGG CGC ACT TT‐3′ and reverse 3′‐TCC ACT GCC CCA CTG AGA A‐5. qRT‐PCRs were carried out with 15 min at 95°C as initial denaturation, followed by 40 cycles including 15 s at 95°C and 1 min at 60°C. After determination of cycle threshold (C_t_) for each reaction, relative gene expressions were measured using 2^‐ΔΔCt^ method as described by Osakabe et al. (Osakabe et al., 2017).

### 2.6 Statistical analysis

One‐way analysis of variance (ANOVA) regarding significant differences (*p* < .05) between means evaluated by Duncan's multiple range test was employed to calculate significant differences among the groups of data using SPSS software version 22.2.5 (SPSS Inc., Chicago, IL, USA). All experiments and measurements were performed in triplicates.

## 3 RESULTS AND DISCUSSION

### 3.1 Effect of SBS on gastric adenocarcinoma cell viability

**FIGURE 1:**
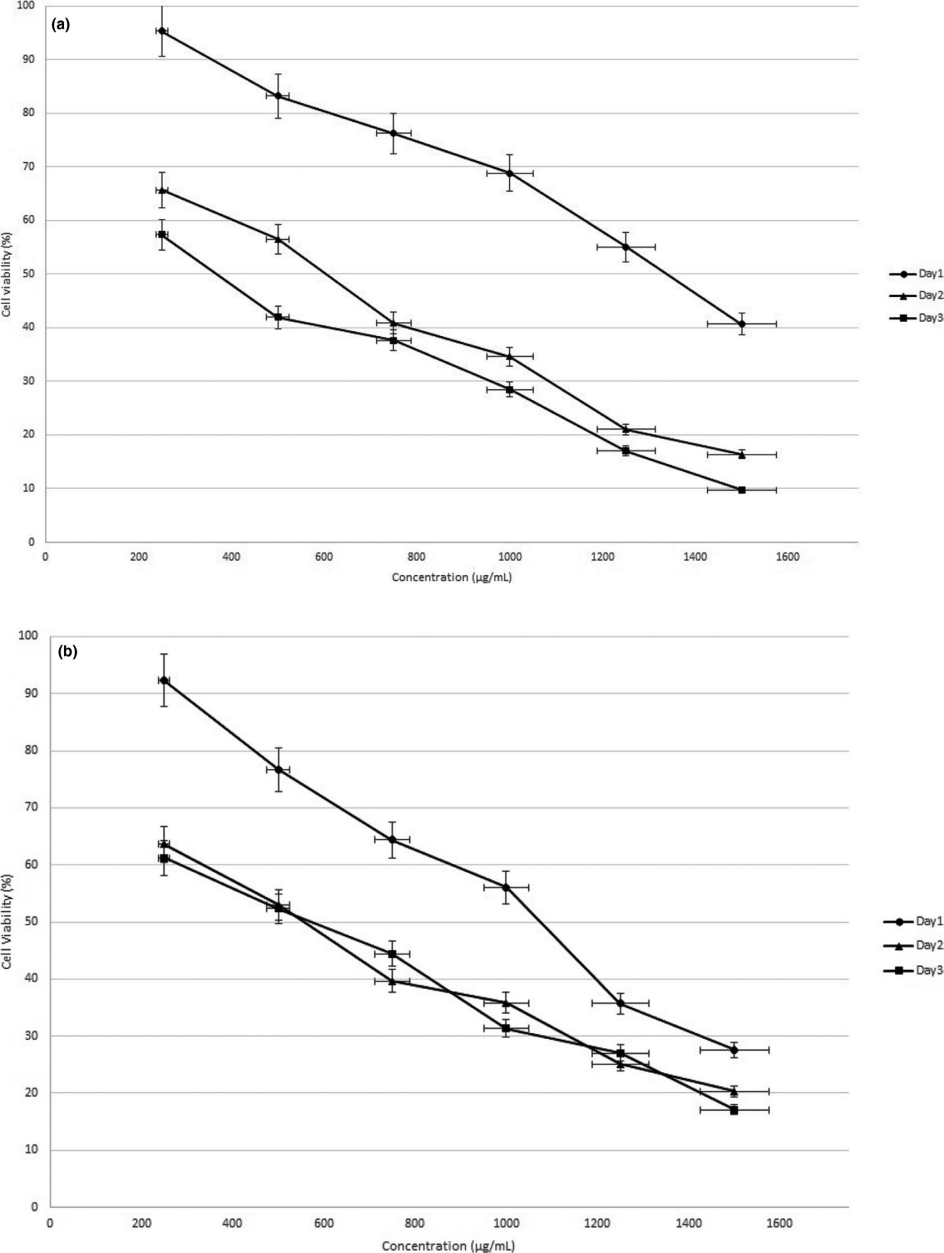
Cell viability of EPG (a) and EPG‐RDB (b) cells under exposure of different concentrations of SBS after 24, 48, and 72 hr by MTT assay

Effect of different concentrations of SBS on cell viability of human gastric cancer cells (EPG) after 24, 48, and 72 hr has been shown in Figure 1a. IC_50_ of SBS after 24‐, 48‐, and 72‐hr treatment was calculated 1,353, 612, and 387 µg/ml, respectively. Figure 1b illustrates the effect of varied concentrations of SBS on daunorubicin resistant human gastric cancer cells (RDB) after 24, 48, and 72 hr. According to Figure 1b, IC_50_ of SBS after 24, 48, and 72 hr was measured 1,044, 575, and 561 µg/ml, respectively. Cytotoxic effect of SBS showed concentration‐dependent, significant (*p* < .05), and considerable decrease in cell viability of both gastric cancer cells (drug‐sensitive and ‐resistant cells). Also, calculated IC_50_ revealed that SBS significantly (*p* < .05) more decreased cell viability of EPG and RDB cells after 72 and 48 hr, respectively, in comparison with other times. Consequently, cytotoxic activity of SBS against human adenocarcinoma gastric cells has been exhibited.

Cytotoxic effect of many natural and synthetic drugs has recently been showed against different types of human cancer cells (Pinto & Silva, 2017). For the first time, Chen et al. (2009) indicated inhibition effect of SBS against growth of intestinal cancer cells and this study was carried out in a mice model. They showed cytotoxic activity of SBS against intestinal cancer cells including HCT‐116, SW480, and HT‐29 cells. They found that SBS significantly reduces the cell viability and proliferation of adenocarcinoma HT‐29 cells after 24 hr (Chen et al., 2009). SBS consist of a complex profile of carbohydrate and protein compounds mostly present in the yeast cell wall of probiotic *S. boulardii*. It is showed that beta glucan and mannan in yeast supernatant prevent growth rate and induce apoptosis in cancer cells (Fortin et al., 2018a, 2018b). Cancer cell antiproliferative activity of yeast cell wall extract is due to insoluble glucans and derived compounds (Choromanska et al., 2015; Sima et al., 2019). In addition to cancer prevention properties, anti‐inflammatory activities of SBS have been known in different in vitro studies such as THP‐1 monocytes, HT‐29 colonocytes, and Caco‐2 cells (Fortin et al., 2018a). SBS also inhibited the proliferation and viability of k562 cancer cell line (myeloid leukemia) (Fatemi et al., 2019). Additionally, anticancer effect of SBS for treatment of colon and breast cancer has been illustrated in rat study models (Chen et al., 2009). Antiproliferative properties were reported for both *S. cerevisiae* and probiotic *S. cerevisiae* var. *boulardii* supernatant (Fortin et al., 2018a, 2018b). It is worthwhile to declare that we observed SBS decreased the cell viability of EPG and RDB cells. We were encouraged to investigate cell apoptosis and survivin gene expression in the case of these cancer cells treated by SBS to complete our study.

### 3.2 Effect of SBS on gastric adenocarcinoma cell apoptosis

**FIGURE 2:**
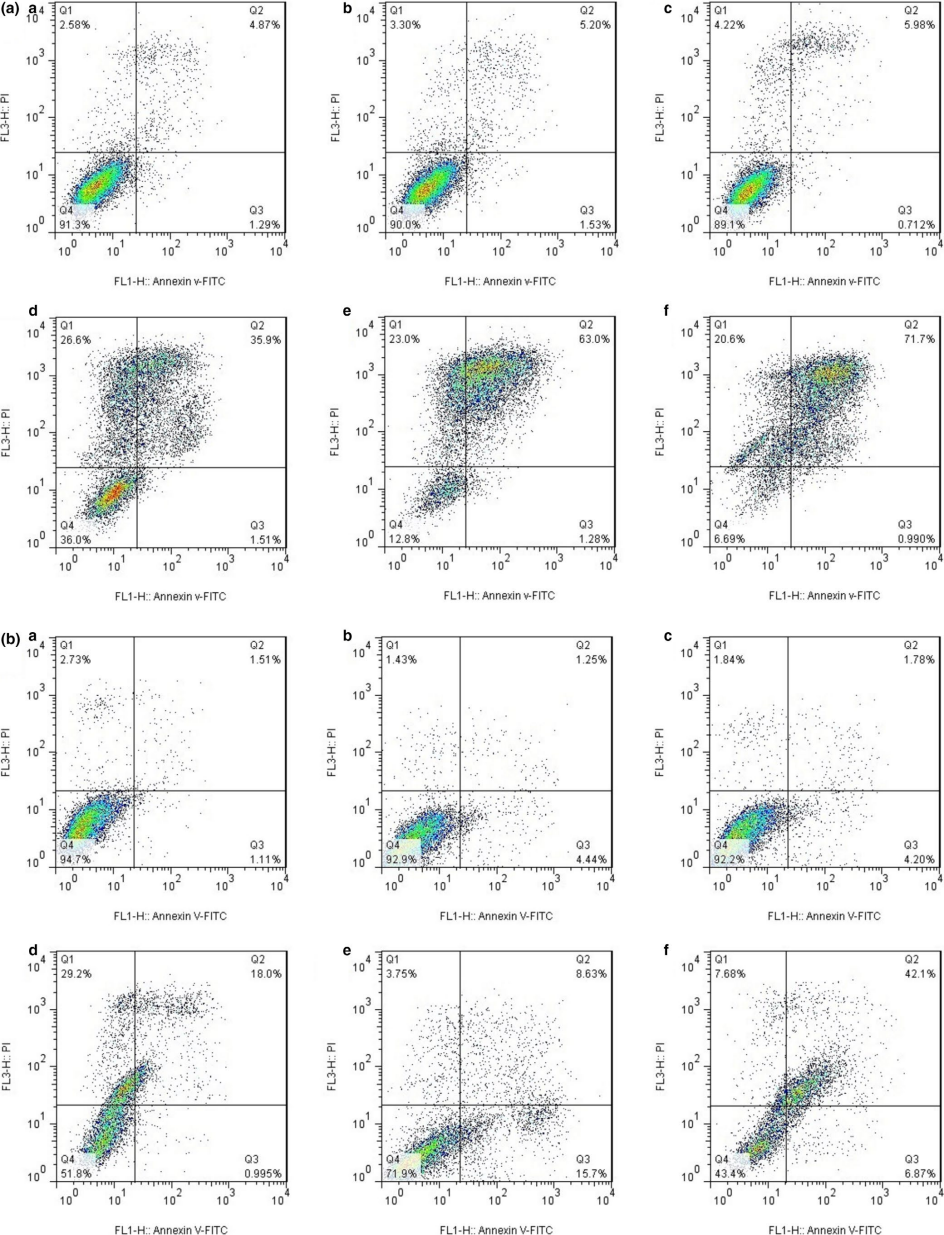
Apoptotic analysis of EPG (A) and EPG‐RDB (B) cells treated with SBS (untreated cells after 24 hr (a), 48 hr (b), and 72 hr (c); and treated cells after 24 hr (d), 48 hr (e), and 72 hr (f)) using flow cytometry method

Results of flow cytometry including Annexin V/PI staining assay for apoptosis analysis of treated and control EPG and RDB cells after 24, 48, and 72 hr are shown in Figure 2a,b, respectively. Proportions of necrotic, late apoptosis, early apoptosis, and live cells were determined in Q1, Q2, Q3, and Q4 quadrants, respectively, in each figure. Total apoptotic cell proportions were calculated by addition of early and late apoptosis proportions (Q2 + Q3) (Hollville & Martin, 2016). Total cell apoptotic proportions were measured less than 7% in all untreated (control) cells after 24, 48, and 72 hr. Cell apoptosis of treated EPG cells was calculated 37.41, 64.26, and 72.69% after 24, 48, and 72 hr; respectively. Also, total apoptotic proportions of treated RDB cells were measured 18.99, 24.33, and 48.97% after 24, 48, and 72 hr; respectively (Table 1). As shown in Figure 2 (a and b), significant (*p* < .05) apoptosis was not observed in untreated cells. All cancer cells treated by SBS showed considerable and significant (*p* < .05) apoptosis after 24‐, 48‐, and 72‐hr treatment. It is also interesting to note that, SBS was able to induce more apoptosis in EPG than RDB cells significantly (*p* < .05).

**Table 1:** Total apoptotic cell proportions and relative survivin gene expression of EPG and EPG‐RDB cells treated with SBS after different time treatment

Time of treatment	Total apoptotic cell proportion (%)		Relative survivin gene expression	
	EPG	EPG‐RDB	EPG	EPG‐RDB
24 hr	31.47 ± 1.12^a^	18.99 ± 3.15^a^	0.618 ± 0.044^a^	0.598 ± 0.012^a^
48 hr	64.26 ± 2.48^b^	24.33 ± 4.22^b^	0.430 ± 0.021^b^	0.494 ± 0.029^b^
72 hr	72.69 ± 1.74^c^	48.97 ± 1.90^c^	0.215 ± 0.038^c^	0.461 ± 0.041^b^

Note

The experimental values within rows having no similar superscript are significantly different (*p* < .05) according to Duncan's multiple test range.

Anticancer drugs induce cytotoxic activity against cancer cells via modulating apoptosis and suppressing cell cycle progression; therefore, researchers measure apoptotic proportions in treated cells to show anticancer activity of drugs (Burke, 2017). Some completely different stimuli including DNA damage, heat shock, reactive oxygen species (ROS), and growth factor depletion lead to activation of apoptotic signals, genes, and cell death (Mohamed et al., 2017). We observed that SBS treatments which more decreased cell viability induce higher cell apoptosis in EPG and RDB cancer cells as it can be interpreted by considering Figures 1 and 2. Xue et al. (2012) investigated anticancer properties of Fucoidan drug on breast cancer in mice and 4T1 mouse breast cancer cells. They found that drug concentrations with higher antiproliferative activity induce more apoptosis in cancer cells (Xue et al., 2012). Apoptosis proportions are associated with the decrease in cell viability for anticancer drug evaluation as we showed at the present study. Purnamasari et al. (2019) found that methanol extract of *Ficus carcia* leaves, as an anticancer drug, decreased proliferation and induced apoptosis of Huh7it cancer cells (Purnamasari et al., 2019). Fang et al. (2019) also introduced neochlorogenic acid an anticancer drug decreased cell viability of AGS as gastric cancer cells and therefore induced apoptosis (Fang et al., 2019).

### 3.3 Change in expression of survivin gene in gastric adenocarcinoma cells after exposure to SBS

**FIGURE 3:**
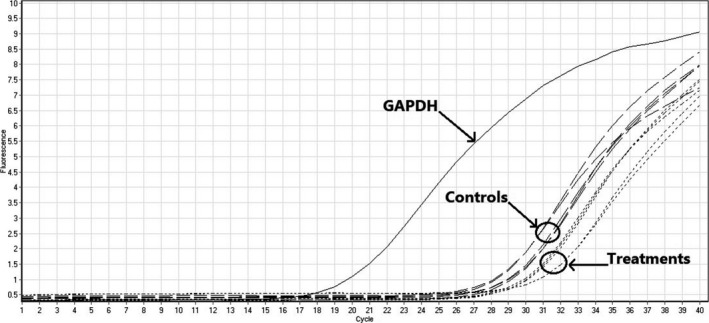
Normalized fluorescence curves of real‐time PCR of survivin mRNA in EPG and RDB cells consisting of GAPDH as the internal control, treated cells with SBS, and control samples treated with DMSO

**FIGURE 4:**
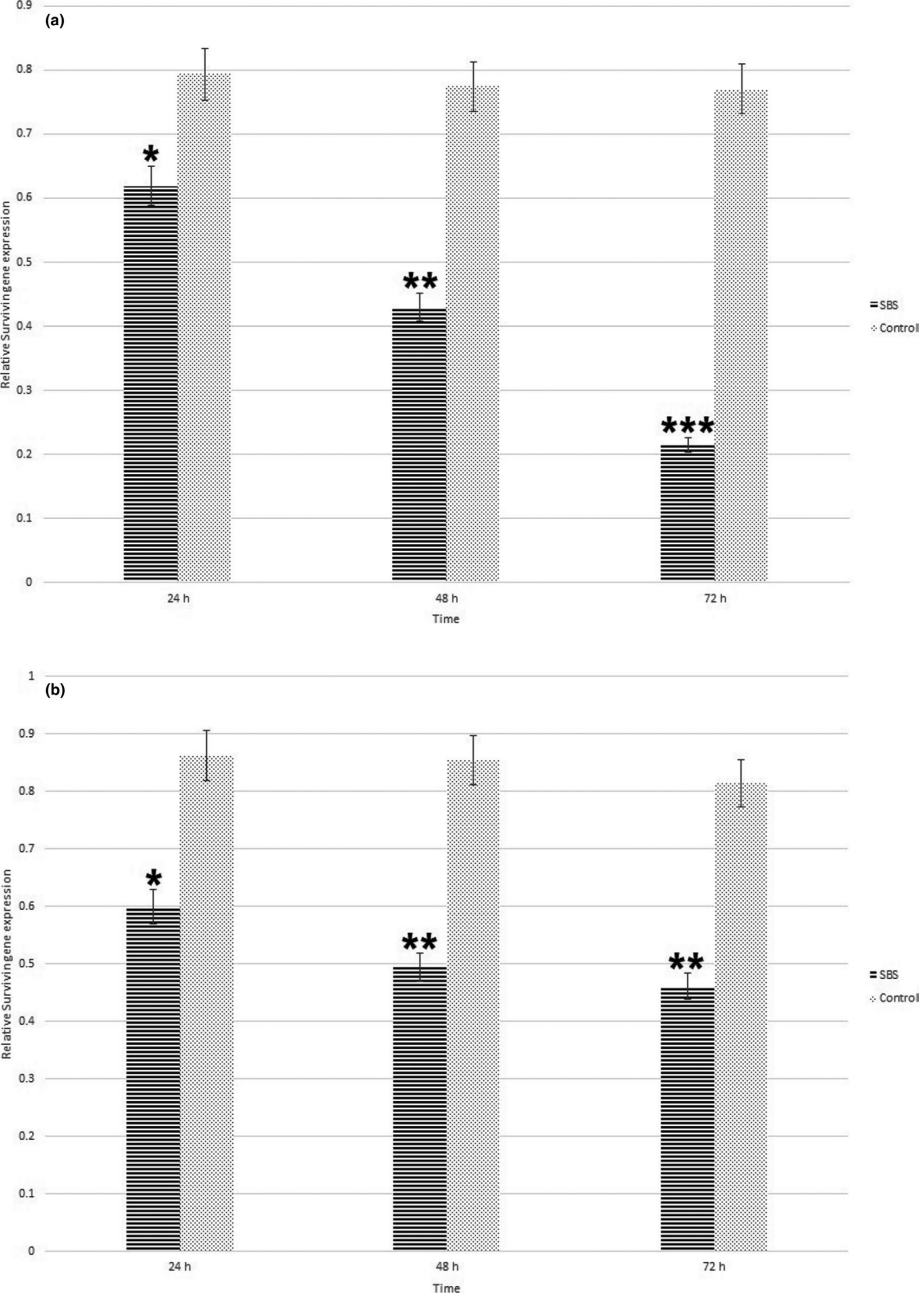
Relative survivin gene expression in EPG (a) and RDB (b) cells including cells treated with SBS and control samples treated with DMSO after 24, 48, and 72 hr. Evaluations were done in triplicate. *, ** and *** indicates significant differences (*p* < .05)

Survivin genes express a group of proteins (BRIC5 protein) preventing cell apoptosis in cancer cells (Wheatley & Altieri, 2019). Anticancer drugs induce cell apoptosis in cancer cells by inhibition of survivin gene expression (Garg et al., 2016). Therefore, at the present study we evaluate anticancer activity of SBS against EPG and RDB cancer cells via measuring change in relative survivin gene expression. We determined changes in relative gene expression by real‐time PCR assay (Figure 3). Relative survivin gene expressions after 24‐, 48‐, and 72‐hr treatment with SBS in EPG and RDB cancer cells were demonstrated in Figure 4a and Figure 4b, respectively. We observed significant (*p* < .05) decrease in relative gene expression in treated EPG cells after 24, 48, and 72 hr; however, our drug reduces the relative survivin gene expression in RDB cells just after 24 and 48 hr. We found that SBS decreased survivin gene expression more in EPG than that in RDB cells (Table 1.). Regarding cytotoxic activity of SBS on both EPG and RDB cells, we found SBS more efficient to decrease cell viability, survivin gene expression, and induce apoptosis of EPG cells. Suppression of survivin gene as the antitumor mechanism of SBS is regarded as one the most important mechanism led to cell apoptosis which has been considered by researchers to in vitro study the anticancer activity of natural and synthetic drugs against cancer cells.

Recently, survivin is the new target of anticancer therapy. Inhibitor of apoptosis protein such as NIAP, XIAP, apollon, and survivin is capable of blocking a major step in cell death progress; therefore, any natural or synthetic compound which prevent survivin activity or expression led to cell death (Garg et al., 2016). One of the prominent known molecular mechanism of anticancer drugs is reduction of survivin gene expression inducing apoptosis and cell death in cancer cells (Martínez‐García et al., 2019). At the present study, we found that prevention of survivin gene expression is one of the anticancer molecular mechanisms of SBS against EPG and RDB cancer cells. Motawi et al. (2014) showed anticancer properties of cromolyn and naproxen against HepG2, Caco2, and MCF7 cells contributed to reduction in survivin gene expression and inducing apoptosis; therefore, it can be used as the complementary medication (Motawi et al., 2014). Oh et al. (2017) declared that downregulation of surviving gene exhibits strongly the anticancer effects of drugs (Oh et al., 2017). RDB cells are gastric cancer cells resistant to daunorubicin which have recently been considered for new drug designing by many researchers (Liu et al., 2017). Borska et al. (2012) also reported higher anticancer activity of quercetin against EPG than RDB cells (Borska et al., 2012). More investigations such as studying signaling pathways, in vivo, and animal model studies are suggested to be implemented to characterize other aspects of anticancer mechanism of SBS against gastric even nongastric cancer cells.

## 4 CONCLUSION

In summary, we provided that yeast supernatant of probiotic *S. boulardii* induces antiproliferative activity, apoptosis, and reduction of survivin gene expression on human gastric adenocarcinoma including EPG and RDB cells. However, we observed SBS with more effective cytotoxic and anticancer activity against EPG than RDB cells. These anticancer properties are due to the presence of a complex profile of glucans and mannoproteins among the cell wall compounds which downregulate the expression of survivin gene and therefore induce apoptosis and cell death progression in cancer cells. SBS may be regarded as a prospective drug or complementary medication to treat or prevent gastric cancer and chronic diseases.

## CONFLICT OF INTEREST

All authors declare that they have no conflict of interest.

## Data Availability

All authors confirm that the data supporting the findings of this study are available within the article.
